# Patterns of perspectives on fall-prevention beliefs by community-dwelling older adults: a Q method investigation

**DOI:** 10.1186/s12877-016-0307-1

**Published:** 2016-07-07

**Authors:** Shueh-Fen Chen, Su-Fei Huang, Li-Ting Lu, Mei-Chuen Wang, Jung-Yu Liao, Jong-Long Guo

**Affiliations:** Department of Senior Citizen Service Management, Ching Kuo Institute of Management & Health, No.336, Fu Hsin Rd., Keelung, 20301 Taiwan; Department of Health Promotion and Health Education, National Taiwan Normal University, No.162, Sec. 1, He-ping East Road, Taipei, 10610 Taiwan; Department of Geriatric Care, Mackay Junior College of Medicine, Nursing, and Management, No.92, Shengjing Rd., Taipei, 11260 Taiwan; Nursing Department, University of Kang Ning, No.137, Lane 75, Sec. 3, Kangning Rd., Neihu District, Taipei City, 11486 Taiwan; Department of Medical Record, Tri-Service General Hospital, National Defense Medical Center, No.325,Sec.2,Chenggong Rd., Neihu District, Taipei City, 11490 Taiwan

**Keywords:** Beliefs, Fall prevention, Q methodology, Older adults

## Abstract

**Background:**

Falling has high incidence and reoccurrence rates and is an essential factor contributing to accidental injury or death for older adults. Enhancing the participation of community-dwelling older adults in fall-prevention programs is crucial. Understanding fall-prevention beliefs will be beneficial for developing a community-based fall-prevention program. The aim of the present study was to identify the distinct types of subjective views on the fall-prevention beliefs of community-dwelling older adults aged 80 years and older by applying the Q method.

**Methods:**

The Q method was adopted to investigate the pattern of perception on fall-prevention beliefs. Forty-two older adults aged 80 − 92 years from a community care center in Northern Taiwan were recruited and requested to complete a Q-sorting. A series of Q-sorts was performed by the participants to rank 30 statements into a normal distribution Q-sort grid. The Q-sorts were subjected to principal component analysis by using PQMethod software Version 2.35.

**Results:**

Four statistically independent perspectives were derived from the analysis and reflected distinct viewpoints on beliefs related to fall prevention. Participants in the Considerate perspective believed that health problems caused by falling were serious and fall prevention could decrease the burden they place on their family. Participants in the Promising perspective believed that existing health problems could cause a fall and that fall prevention contributed to their well-being. Participants in the Adaptable perspective perceived low barriers to execute fall prevention and displayed self-confidence and independence in preventing falls. Participants in the Ignorance perspective believed that they could not prevent falls and perceived barriers to fall prevention.

**Conclusions:**

By combining theoretical constructs and the Q methodology approach, this study identified four distinct perspectives on fall prevention among community-dwelling older adults. Critical reflection on older adult personal perspectives and interpretations of the required responsive approach is a key element for appropriating fall-prevention support.

**Electronic supplementary material:**

The online version of this article (doi:10.1186/s12877-016-0307-1) contains supplementary material, which is available to authorized users.

## Background

Falling has high incidence and reoccurrence rates and is an essential factor contributing to accidental injury or death for older people [1–3]. Prevalence rates for falls in community-dwelling older adults range from 17.2 % to 33.1 %, and reoccurrence rates are 5.7 % to 15.2 % [2, 4, 5]. Falls are particularly prevalent in older adults aged 80 years and older, and the proportion of this population experiencing falls is increasing more sharply than that of other age groups; fall rates can be as high as 50 % per year [2, 6, 7]. Falls can result in injuries, including physical and mental, and are related to family and social effects. These are devastating problems causing major morbidity and mortality among older people, particularly older adults aged 80 years and older [6, 7]. Falls lead to severe outcomes that not only impose a considerable financial burden on families but also increase associated social and health care costs [8]. Therefore, strategies and methods for preventing or reducing the incidence of falls in older adults have become a crucial topic [8, 9].

Systematic reviews and meta-analyses revealed that promoting appropriate physical activities or exercises to improve strength, balance, and flexibility is one of the most feasible and cost-effective strategies to prevent falls among older adults in the community [2, 4, 5]. A critical difficulty in obtaining the benefits of fall-prevention activities is to motivate older adults to actively participate in the activities [2]. However, older adults may not regularly participate in fall-prevention intervention programs. Studies indicated that older adult participation rates are not high: Approximately 40 % of people aged 60 years and older have not reached the minimum recommended level [10, 11]. If older adults perceive falls as a normal consequence of ageing expressed as “seniors will always fall”, their attitudes may halt preventive behaviors [2]. Research indicated that health professionals should consider the subjective views of older adults to trigger their participation in fall-preventive activities [12].

Older adult perceptions play a vital role in their willingness to engage in fall-prevention activities [13]. Understanding fall-prevention beliefs of older adults contributes to developing successful community-based fall-prevention interventions [14]. Simons [15] indicated that the Q methodology is frequently used to investigate individual subjective attitudes because it enables interviewees to smoothly express their views through statements. An evidence revealed that using the Q methodology for investigating subjective views facilitates communication for people who experience difficulty expressing their views [16]. The methodology provides a logical process for systematically investigating the subjective views, opinions, beliefs, and attitudes of participants. It was used successfully to investigate the diverse health concerns in health promotion [17].

We found that the four health belief model (HBM) constructs (susceptibility, severity, benefits, and barriers to taking action) coincided with the background of this study and were suitable as a theoretical guideline for developing Q-statements for assessing individual beliefs in preventive fall. Previously, researchers adopted the HBM to explore the views of community-dwelling older adults; it was used to verify and elucidate preventive health behaviors [18, 19]. As previously mentioned, the risk of falling was strongly associated with older adults aged 80 years and older. Thus, the aim of the present study was to identify the distinct types of subjective views on the fall-prevention beliefs of community-dwelling older adults aged 80 years and older by applying Q methodology.

## Methods

### Participants

The study targeted community-dwelling older adults from community care centers in a city in Northern Taiwan. The community care centers provide health promotion services through voluntary sectors under the government subsidies. Community-dwelling older adults can participate in the health promotion services gratuitously. The inclusion criteria of participants were as follows: (a) being more than 80 years old, (b) able to express their views in Mandarin or Taiwanese, and (c) willingness to participate in the study and sign an informed consent.

A previous study suggested that the Q methodology was not designed for hypothesis testing; thus, the sample size was not estimated [20]. The Q methodology is a type of exploratory factor analysis. Diverse viewpoints would be achieved most effectively with a group of 40–60 participants [21]. On the basis of this suggestion, we recruited 50 older adults aged 80 or older who agreed to participate in the study, and 42 older adults successfully completed the Q-sorting.

### Demographic information

Demographic information, including age, gender, education level, marital status, living status, fall experience (last year), and cardiovascular disease, was collected using a structured questionnaire.

### Procedure

Applying the Q methodology involved compiling the Q-sorting data derived from the Q-statements sorted by the interviewees. Subsequently, a Q-perspective analysis was conducted to classify the interviewees and explore their characteristics. The Q-sorting facilitated presenting and thereby clarifying the interviewee subjective views on the Q-statements, thus elucidating their attitudes toward fall prevention. The Q method procedure is described as follows:

#### Phase I. Developing a concourse and constructing Q-statements

Developing a concourse is the first step in the Q methodology. A concourse refers to a summary of opinions related to interesting issues. A theoretical concept based on the HBM [18] was adopted to compile fall-prevention experiences and views from the perspectives of community-dwelling older adults. Q-sorting items were referred to literature reviews and views collected from six focus group interviews on community-dwelling older adults [22]. We obtained a total of 73 statements that constituted the concourse used in this study. The content validity of the 73 statements was assessed by two experts in older adult health promotion and fall prevention to secure each statement distinctly and plainly. Ambiguous and confusing statements were eliminated or modified to ensure that they were comprehensible to the interviewees. Throughout a series of consultations, the research team finally revised and reduced the statements to a set of 30 items. Eight participants were recruited for the pilot study to test the validity and reliability of the items. Ambiguous and confusing statements were remodified to ensure that the interviewees comprehended the statements. The final set of the 30 Q-statements were representative of the original “written views or sayings” about fall prevention provided by the members of the focus group.

#### Phase II. Administering the Q-sorts

**Fig. 1:**
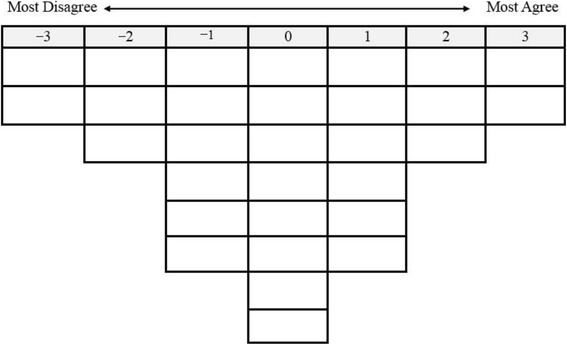
Forced-choice frequency distribution in Q-sort

The implementation of the Q method involved a Q-sorting process performed by the interviewees. Each older adult was visited individually by a trained interviewer who conducted the interview at a quiet room of the community care center. The 30 Q-statements were separately printed on a card in advance; these cards were called Q-samples. The interviewee was requested to sort and rank these cards by following a procedure. Verbal instructions regarding how to complete the Q-sorting were provided. The trained interviewer shuffled the cards prior to administration, and the participants were advised to read the statements and rank the items by placing the card in a sorting grid depicted in Fig. 1. The trained interviewer read the cards out loud for those who could not read by themselves. Each column of the grid represented a response from +3 (*most agree*) to −3 (*most disagree*). Each set of the ranked grid was collected for each participant. The participants were allowed to move statements during the sorting process until they felt comfortable with the distributions of all statements. The final grid comprised the participant Q-sorting, which was placed on a data collection paperboard and photographed by the interviewer. The participants spent approximately 30 min to complete the Q-sorting.

### Data analysis

The statistical software package PQMethod Version 2.35 was employed to analyze the Q-sorting data (Additional file 1). The Q methodology is typically used for exploratory research. Therefore, principal component analysis with Varimax rotation was conducted to extract relevant perspectives. Regarding the classification principle, Schinger [23] recommended that perspective loadings be greater than 2.58/√i to demonstrate significance (i is the number of statements). Consequently, in this study, only if the perspective loadings were greater than 2.58/√30 = 0.47 and presented as the highest, then they served as the classification criterion. A combination of eigenvalues that reflected the amount of variation accounted for by a corresponding factor and a scree plot was employed to determine the number of retained factors. Finally, four perspective factors with eigenvalues greater than one were derived. We determined that the four-factor solution was the “best-fit” for the data. Each factor comprised at least three Q-sort loadings that were high and significant (*p* < 0.05) on only one factor.

## Results

A total of 42 participants aged 80–92 years (84.05 ± 3.18 years) were recruited; 76.2 % (*n* = 32) of them were women, 31.0 % (*n* = 13) had experienced a fall in the previous year, 47.6 % (*n* = 20) were illiterate, 61.9 % (*n* = 26) were single (i.e., unmarried, divorced, or widowed), 19.0 % (*n* = 8) lived alone, and 66.7 % (*n* = 28) had a history of cardiovascular disease. Four perspective factors were derived from the Q-sorting results (Table 1). The explained variance by the four perspectives were 36.05 % (eigenvalue = 15.14), 9.52 % (eigenvalue = 4.00), 7.93 % (eigenvalue = 3.33), and 6.29 % (eigenvalue = 2.64). The total explained variance was 59.79 %. All demographic variables were stratified by the four perspective factors (Table 2).

**Table 1:** The factor scores of participants associated with the four factors (*n* = 42)

Participant No.	Factor 1 (*n* = 14)	Factor 2 (*n* = 13)	Factor 3 (*n* = 12)	Factor 4 (*n* = 3)
36	0.8286	0.3661	0.0615	−0.0998
18	0.8210	0.2257	0.0710	0.1997
17	0.7638	0.2048	0.2408	0.1817
29	0.7220	0.3595	0.1199	0.1315
13	0.6924	0.2232	0.1726	−0.1621
10	0.6844	0.0856	0.2962	0.2043
06	0.6544	0.0936	0.2130	−0.1571
11	0.5954	−0.1311	0.1254	−0.1458
34	0.5818	0.3785	0.2026	0.5606
42	0.5244	−0.1151	0.2669	0.3938
09	0.5221	0.1080	0.2486	−0.1835
15	0.5178	−0.1154	0.3232	−0.0454
20	0.4957	0.4904	−0.2400	0.1287
26	0.4940	0.3127	0.4415	−0.1275
37	−0.0859	0.7911	−0.1652	0.1758
32	0.0994	0.7614	0.3271	0.0684
38	0.2150	0.7387	0.2646	−0.1430
04	0.0145	0.7278	0.0457	−0.0325
27	0.2394	0.6792	0.3957	0.0446
22	0.3456	0.6496	0.2222	0.1021
39	0.3854	0.6014	0.2162	−0.1183
31	0.3680	0.6014	0.3851	0.1736
25	−0.1350	0.5817	0.1554	−0.0465
05	0.3439	0.5561	−0.1346	−0.1303
08	0.1379	0.5382	0.1975	0.1344
30	0.2957	0.5354	0.4067	0.0003
14	0.0066	0.5121	0.4855	−0.2681
41	0.2793	0.1937	0.7778	−0.0313
33	−0.2669	0.1654	0.7668	0.0508
40	0.2495	0.1305	0.7548	0.1055
07	0.2200	0.1447	0.6756	0.3120
01	0.4394	0.2386	0.6682	0.0099
03	0.4526	0.0412	0.6298	0.0912
35	0.0910	0.4451	0.6098	−0.2217
02	0.4575	0.3047	0.6028	0.3366
16	0.3863	0.1960	0.5695	−0.0615
12	0.4360	0.3026	0.5593	0.2297
24	0.2623	−0.0248	0.5572	0.3214
28	0.4146	0.1774	0.5393	0.4042
23	−0.0781	0.1555	−0.2002	0.7430
19	−0.0564	−0.1956	0.1424	0.6675
21	−0.0968	0.0048	0.2344	0.6406
Eigenvalue	15.14	4.00	3.33	2.64
Explained Variance (%)	36.05	9.52	7.93	6.29

**Table 2:** Socio-demographic characteristics with the four factors

Variables	F1. Considerate (*n* = 14)	F2. Promising (*n* = 13)	F3. Adaptable (*n* = 12)	F4. Ignorant (*n* = 3)
Age (y, Mean ± SD)	82.09 ± 2.40 (80 − 88)	85.38 ± 2.76 (80 − 91)	84.58 ± 3.02 (81 − 89)	84.67 ± 6.35 (81 − 92)
Gender				
Female	13 (92.9 %)	10 (76.9 %)	7 (58.3 %)	2 (66.7 %)
Male	1 (7.1 %)	3 (23.1 %)	5 (41.7 %)	1 (33.3 %)
Education				
Illiteracy	3 (21.4 %)	7 (53.8 %)	9 (75.0 %)	1 (33.3 %)
Primary school	9 (64.3 %)	4 (30.8 %)	2 (16.7 %)	1 (33.3 %)
Middle school or higher	2 (14.3 %)	2 (15.4 %)	1 (8.3 %)	1 (33.3 %)
Marital status				
Single	10 (71.4 %)	7 (53.8 %)	7 (58.3 %)	2 (66.7 %)
Married	4 (28.6 %)	6 (46.2 %)	5 (41.7 %)	1 (33.3 %)
Living status				
Solitary	3 (21.4 %)	2 (15.4 %)	3 (25.0 %)	0 (0 %)
Non-solitary	11 (78.6 %)	11 (84.6 %)	9 (75.0 %)	3 (100 %)
Fall experience (last year)				
No	11 (78.6 %)	7 (53.8 %)	9 (75.0 %)	2 (66.7 %)
Yes	3 (21.4 %)	6 (46.2 %)	3 (25.0 %)	1 (33.3 %)
Cardiovascular disease				
No	1 (7.1 %)	3 (23.1 %)	8 (66.7 %)	0 (0 %)
Yes	13 (92.9 %)	10 (76.9 %)	4 (33.3 %)	3 (100 %)

### Consensus about falling and fall prevention

Among the 30 Q-statements employed in this study, Q-statement 10, “Falls may cause bleeding, external injury, bone injury, spinal injury, or brain injury” (2, 2, 3, 3) was rated agree the most among the four perspectives. The values in the parentheses represent the Q-sort values for the four perspectives, indicating how falls could incur bone, spinal, or brain injury could be a crucial belief that affected the participant attitudes toward fall prevention. Q-statement 15, “After experiencing a fall, I might not be able to walk (requiring the support of a wheelchair) and a health care worker might be hired to look after me, thus creating burdens for my family” (3, 3, 3, −2) was rated as agree the most among the first three perspectives, indicating how the inability to walk incurred burdens to family members could a crucial belief influencing most of the participant attitudes toward fall prevention. Examining these two Q-statements revealed that the main belief affecting the attitudes of most participants toward fall prevention was the seriousness of a fall. In addition, Q-statement 5, “I feel that when I am in a bad mood, I am easily distracted and became careless, which might cause me to fall” (−2, 0, −3, −3) was rated as disagree the most among three of the perspectives. Q-statement 27, “I cannot prevent myself from falling because I live upstairs and need to climb the stairs every day” (−3, −2, −1, −1) was rated as disagree among all the perspectives. Therefore, a participant mood and need to climb the stairs at home were not prevailing fall-prevention beliefs among most participants. The Q-sort values and characteristics of the participants associated with the four perspectives are described in Table 3.

**Table 3:** List of Q-statements and factor Q-Sort values associated with the four Perspectives

Q-Statement	F1. Considerate	F2. Promising	F3. Adaptable	F4. Ignorant
01. I believe that I am old now and often react slowly, which might make me accidentally bump into objects and stumble	1**	0	0	−1
02. I believe that sudden dizziness, brain disease, and cardiovascular disease can cause a loss of physical imbalance, which could lead to a fall	−3**	3	2	1
03. I believe degenerative joint diseases could make me fall	−2	−2	1	1
04. I believe that deteriorating eyesight could make me fall	0**	−3	1**	−3
05. I feel that when I am in a bad mood, I am easily distracted and became careless, which might cause me to fall	−2	0**	−3	−3
06. I believe that wet and slippery floors (indoors or in a bathroom) can make me fall over	1	−1**	1	1
07. I believe that rough roads and road obstacles can make me fall	−1	−1	−1	0
08. I believe that going out at night and insufficient light can make me fall	−1	−3	−1	−1
09. Falls may cause bruise and sprain	1	0	2	1
10 Falls may cause bleeding, external injury, bone injury, spinal injury, or brain injury	2	2	3	3
11. Falls may cause joint dislocation and bone fracture.	1**	2	2	−1**
12. Falls may make me reluctant to move, resulting in physical function degeneration and disability	2**	1	1	−2**
13. After experiencing a fall, I might be afraid of experiencing another fall	0	1**	0	0
14. After experiencing a fall, I might encounter life problems such as physical pain, discomfort, and inconvenience	3	1	1	−1**
15. After experiencing a fall, I might not be able to walk (requiring the support of a wheelchair) and a health care worker might be hired to look after me, thus creating burdens for my family	3	3	3	−2**
16. I believe that fall prevention can reduce the occurrence of fall accidents	0	1**	0	0
17. I believe that fall prevention enhances my well-being	0	2**	0	0
18. I believe that fall prevention can give my family members peace of mind	1**	0	−1	0
19. I believe that fall preventive can reduce my family of burdens	2	0	−1	1
20. I believe that fall prevention can make people close to me happy	1	0	−1**	1
21. I cannot prevent myself from falling because I am clumsy and stumble easily	−1	−1	0**	2**
22 I cannot prevent myself from falling because I am old and stubborn	−2	0	−2	−1
23. I cannot prevent myself from falling because I do not want to trouble other people (acting strong)	0	0	−3**	3**
24. I cannot prevent myself from falling because of hurriedness when hearing doorbell or phone ring	−1	1	−2	0
25. I cannot prevent myself from falling because I cannot foresee when an accidental fall is going to occur (i.e., accidents happen because other people do not pay attention)	−1	−1	0	2**
26. I cannot prevent myself from falling because I do not have sufficient prevention knowledge and skills	0	−2	−2	1
27. I cannot prevent myself from falling because I live upstairs and need to climb the stairs everyday	−3	−2	−1	−1
28. I cannot prevent myself from falling because I am not aware arcades or rough roads	0	−1	0	0
29. I cannot prevent myself from falling because of dim light	0	−1	0	−2
30. I cannot prevent myself from falling because of few installation of handrails in bathroom, stairs and infrastructures	−1	1	1	0

Note:1. The statements were written down in plain language to make the less-educated older adults to comprehend

2. Factor Q-sort values were identified by Q-sort factor analysis and indicated that the statements ranked from +3 (*most agree*) to -3 (*most disagree*)

3.** Significance of Distinguishing Statements *P* < 0.01

### Factor 1: Considerate perspective

We named this perspective the Considerate perspective because the participants who adhered to this view recognized the fall-related injury and burden to their family; therefore, fall prevention could decrease the burdens they placed on their family. For example, this perspective revealed a strong concern on fall-related life problems such as statement 14 (Q-sort value, Z-score: 3, 1.427). Older adults with the Considerate perspective believed that fall prevention could decrease the burdens they place on their family (2, 0.885).

Regarding Q-statements, the main distinguishing statements for this perspective compared with the counterparts are as follows: “Falls may make me reluctant to move, resulting in physical function degeneration and disability” (2, 1.415); “I believe that I am old now and often react slowly, which might make me accidentally bump into objects and stumble” (1, 0.758); “I believe that fall prevention can give my family members peace of mind” (1, 0.691).

Among the 14 participants loaded significantly on this factor, the average age (82.09 ± 2.40 years) was the youngest among the four groups. Most of them were women (13, 92.9 %) (except for one participant). Participants associated with the Considerate group had the highest percentages of suffering from cardiovascular diseases (13, 92.9 %), being single (10, 71.4 %), and with education (11, 78.6 %) compared with the other groups (Table 2). They were considerate about fall prevention to prevent them from becoming burdens to their family.

### Factor 2: Promising perspective

In this perspective, which we named the Promising Perspective, the participants classified into this perspective believed that sudden dizziness, brain disease, and cardiovascular disease could cause physical imbalance, leading to a fall (3, 1.472); falls might cause joint dislocations or bone fractures (2, 1.189). However, they also believed that fall prevention contributed to their well-being (2, 1.468); they did not believe that they lack prevention knowledge and skills that prevent them from falling (−2, −1.220).

The main distinguishing statements for the participants compared with the counterparts are as follows: “I believe that fall prevention enhances my well-being” (2, 1.468); “I believe that fall prevention can reduce the occurrence of fall accidents” (1, 0.698). These participants believed that they could perform well in facing risks of falling such as “I believe that wet and slippery floors (indoors or in a bathroom) can make me fall over” (−1, −1.113).

Among the 13 participants loaded significantly on this factor, whose average age was 85.38 ± 2.76 years, nine were older than 85 years, which comprised the highest percentage among the four groups. Participants associated with the Promising group had the highest percentages of married status (6, 46.2 %) and fall experiences during the last year (6, 46.2 %) compared with the other groups (Table 2). These participants believed that multiple health problems could cause them to fall, but physical degeneration or environmental barriers would not impair their ability to prevent a fall.

### Factor 3: Adaptable perspective

We named this perspective the Adaptable perspective. The participants who adhered to this perspective felt that health problems resulted in a fall. They believed that sudden dizziness, brain disease, and cardiovascular disease could cause physical imbalance, resulting in a fall (2, 1.121). In addition, they believed that negative health outcomes of falls were very serious: bruises and muscular and skeletal injuries (2, 1.280), as well as joint dislocations and bone fractures (2, 1.131). However, they disagreed the most that the following reasons could impair their fall-prevention efforts: They did not want to trouble other people (acting strong) (−3, −1.797); they were old and stubborn (−2, −1.486); they hurried when they heard the doorbell or the phone ringing (−2, −1.305); and their knowledge and skills on fall prevention were inadequate (−2, −1.120).

Regarding fall-prevention beliefs, the main distinguishing statements for the participants compared with the counterparts are as follows: “I cannot prevent myself from falling because I do not want to trouble other people (acting strong)” (−3, −1.797); and “I believe that fall prevention can make people close to me happy” (−1, −0.775). These participants believed that they faced the risks of falling and would like to use their own knowledge and skills to manage and receive support from other people.

Among the 12 participants loaded significantly on this factor, the average age was 84.58 ± 3.02 years. Compared with the other groups, participants associated with the Adaptable group had the highest percentages of being male (5, 41.7 %), illiteracy (9, 75.0 %), and living alone (3, 25 %) and the lowest percentage of suffering from cardiovascular diseases (Table 2). These participants were confident in preventing falls and did not believe that fall prevention would benefit their family or other people close to them.

### Factor 4: Ignorant perspective

Participants from the Ignorant perspective expressed external locus of control and a low perception of risks of falling, and demonstrated low levels of self-efficacy and efforts on preventing themselves from falling. For example, they believed that they could not predict when an accidental fall was going to occur (2, 1.309); they could not prevent themselves from falling because they did not want to trouble other people (3, 1.554), and they were clumsy and stumbled easily (2, 1.432). Participants in this group disagreed the most that deteriorating eyesight could cause them to fall (−3, −1.322), and they did not believe that the following situations were serious: After experiencing a fall, they would experience physical function degeneration and disability because of their reluctance to move (−2, −1.309); following a fall, if they could not walk (or required the support of a wheelchair), then they might need a health care worker to look after them, thereby incurring a burden on their family (−2, −1.224). Additionally, they did not believe that walking in dimly lit areas would cause them to fall (−2, −1.127).

The main distinguishing statements for the participants with this perspective compared with the counterpart are as follows: “I cannot prevent myself from falling because I do not want to trouble other people (acting strong)” (3, 1.554); “I cannot prevent myself from falling because I am clumsy and stumble easily” (2, 1.432); “I cannot prevent myself from falling because I cannot foresee when an accidental fall is going to occur” (2, 1.309); “Falls may make me reluctant to move, resulting in physical function degeneration and disability” (−2, −1.309); “After experiencing a fall, I might not be able to walk (requiring the support of a wheelchair), and a health care worker might be hired to look after me, thus creating burdens for my family” (−2, −1.224); and “After experiencing a fall, I might encounter life problems such as physical pain, discomfort, and inconvenience” (−1, −0.490).

Only three participants, including the oldest interviewee in this study (92 years old), were loaded to the Ignorant perspective. All of the participants were non-solitary and with cardiovascular diseases (Table 2). These participants demonstrated the attitude of ignorance regarding the risks and negative outcomes of falling. In addition, they believed that fall-prevention barriers comprised of clumsiness, not wanting to trouble other people, and an accidental fall that could not be prevented.

## Discussion

In this study, the Q methodology was adopted to analyze various fall-prevention beliefs among community-dwelling older adults aged 80 years and older, and to elucidate the four distinct perspectives. The main differences between the four perspectives were related to the perceived severity, benefits, and barriers.

Community-dwelling older adults, the target population in the study, were healthier, more independent, and more moveable compared with their institutionalized counterparts [24]. The main consensus among them was that they recognized that fall-related injuries were serious and might cause disability, multiple health complications, and place burdens on their family; these are consistent with the prior findings [1, 3, 25]. However, they did not believe that bad mood, such as depressed status and environmental risk factors, were barriers that prevent themselves from falling; this result was not consistent with the prior evidence [26, 27].

Compared with the counterparts with different perspectives, the participants with the Ignorant perspective demonstrated a low level of perception on severity. They tended to underestimate the risks and outcomes of falling. They were less moveable but would like to act strong, indicating a low level of perception on severity. They believed that they were clumsy and stumbled easily, and an accidental fall was not predictable. Moreover, they did not believe that after experiencing a fall, they would face negative health outcomes and inability and become a burden to their family. Compared with the counterparts with various perspectives, they perceived a high level of barriers to execute fall-prevention activities. Inadequate beliefs may make them more likely to fall in the near future; fall-prevention practitioners, family, and caregivers should work together to provide educational opportunities, environmental safety check, and protective devices [28, 29]. The participants with the Ignorant perspective should be encouraged to participate in fall-prevention activities and regard it as a high priority. Fall-prevention practitioners are suggested to motivate elderly adults to continue participating in preventive activities, modify their unfavorable beliefs, and provide individualized supports.

The characteristics of the participants with the Considerate perspective were that most of them were women, had cardiovascular diseases, and were tied to the family. A distinct belief of these participants was that they were concerned about family and would not like to place burdens on their family. They believed that the outcomes of falling, such as daily life inconvenience and physical injury, were very serious, and they were concerned that falling would cause them to develop a disability, making them a burden to their family; hence, these considerations motivated them to prevent falling. The implications of enhancing participation in fall-prevention program for these considerate participants were to continue emphasizing that risk factors were preventable and the benefits of participation, raising their level of self-efficacy [14, 17], and encouraging their family to provide social supports when they participate in fall-prevention activities.

Participants with the Promising perspective expressed similar and distinct beliefs with those with the Considerate perspective. They both believed the susceptibility of falling but in different dimensions (Table 3), and they shared similar beliefs on the severity of fall-related injuries. Regarding the benefits of fall prevention, they were more likely to believe that it was advantageous for themselves, not for their family or the people close to them. They were more active and independent to participate in fall-prevention activities. Their characteristics were as follows: they exhibited the highest average age, had multiple health problems, and were willing to take actions to enhance their personal well-being (Table 3). The implications of encouraging them to continue participating in fall-prevention programs were to provide reminders and incentives for participation, which are appropriate strategies of cueing them to take action.

The participants with the Adaptable perspective perceived their susceptibility to the severity of falling. The characteristics of these elderly participants compared with the other counterparts were as follows: they were healthier, more independent, and had higher levels of self-efficacy to overcome barriers, allowing them to receive support from people close to them. Compared with the other counterparts, they did not believe that the benefits of fall prevention were to give their family members peace of mind, reduce family burdens, and make the people close to them happy. They perceived low barrier levels to engage in fall-prevention activities. Fall-prevention practitioners suggested asking them to be the demonstrators during the implementation of fall-prevention activities and role models on fall prevention [30].

The strength of our study is that we conducted a pilot test to modify the Q-statements and made it more readable to the participants. We provided a large paperboard to represent each statement and advice on how to execute Q-sorting correctly. Thus, we successfully classified all participants into four factors with satisfactory loading scores. The total explained variance was 59.79 % in this study, although some researchers used the guideline of retaining enough factors to account for more than 75 %–90 % of the variance [31, 32]; others indicated that accounting for 50 % of the variance was acceptable [33].

We used purposive sampling, inviting representatives from different genders, education levels, marital status, living status, fall experiences, and cardiovascular diseases with different perspectives on fall prevention. Consequently, we had diverse participants with the same characteristics of living in communities, which were likely to produce four distinct perspectives on fall prevention. However, we may not have gathered all the existing beliefs of community-dwelling older adults toward fall prevention. All participants lived in the same city; however, older adults from other geographical areas may differ over health beliefs toward fall prevention. The next step would be to conduct a Q-methodological survey among a larger sample of older adults including those who are institutionalized.

## Conclusion

This study incorporated both the theoretical concepts and the Q methodology approach into the study design and provided approaches to identifying four distinct perspectives of community-dwelling older adults on fall prevention. The older adults in the Ignorant perspective appeared to be a preferred subpopulation to be recruited for fall-prevention activities. According to which perspective the older adult participants of fall-prevention activities belong, fall-prevention practitioners, family members, and caregivers should be aware of what kind of approach may be required to encourage them to respond. Critical reflections on elderly adult own perspectives and interpretations of the responsive approach required in a certain situation are a key element for appropriating fall-prevention support. The study highlights that fall-prevention programs are suggested to be designed in the context of physical, psychological, and familiar demands derived from the various beliefs of older adults to improve their participation in and adherence to program activities.

## Abbreviation

HBM, health belief model

## Additional file

Additional file 1: Q-sorting data. (DOCX 14 kb)
