# Supplementary material for: Patterns of perspectives on fall-prevention beliefs by community-dwelling older adults: a Q method investigation
Source: BMC Geriatr. 2016 Jul 7;16:132. doi: 10.1186/s12877-016-0307-1 (PMC4936088; doi:10.1186/s12877-016-0307-1)
Supplement: Additional file 1: — Q-sorting data. (DOCX 14 kb) [file 12877_2016_307_MOESM1_ESM.docx]

**Additional file 1. Q-sorting data**

0 42 30 oldfall

1 7 0 0 0 0 0 0 0 2 3 6 8 6 3 2 0 0 0 0 0 0

n01 5 3 5 6 3 4 2 2 5 7 6 5 3 5 7 4 5 4 4 4 4 1 1 4 4 3 3 2 3 6

n02 3 4 4 5 1 6 3 3 6 7 6 5 5 3 7 5 5 4 4 4 5 1 2 4 4 2 2 3 3 4

n03 6 4 3 6 1 5 4 3 5 7 5 5 2 5 7 3 4 3 4 3 5 4 3 2 6 1 2 4 4 4

n04 5 6 3 2 4 1 3 2 3 4 4 4 5 5 7 6 7 3 5 3 4 5 4 4 3 4 1 5 2 6

n05 5 2 3 1 3 2 3 2 6 5 7 4 6 7 4 4 5 5 4 6 1 5 3 5 3 3 4 4 4 4

n06 6 1 5 4 4 4 2 4 5 5 3 6 7 6 7 5 4 3 4 3 4 2 2 3 3 4 1 5 5 3

n07 3 7 5 6 1 4 3 3 5 5 5 3 3 6 7 4 4 4 5 4 6 2 4 2 4 1 2 5 4 3

n08 3 7 4 4 3 1 3 2 4 6 4 6 4 2 7 5 5 5 5 6 2 5 3 3 5 4 1 4 4 3

n09 6 1 2 5 4 3 6 3 6 7 4 5 4 4 7 2 3 5 4 3 4 5 2 4 4 3 1 5 3 5

n10 4 1 4 3 1 5 5 3 6 7 6 5 2 5 6 4 4 4 5 7 3 3 3 2 4 2 3 5 4 4

n11 6 1 5 6 2 4 2 3 4 3 7 4 3 6 7 4 4 2 5 5 3 3 4 4 5 5 4 5 3 1

n12 3 6 4 4 2 4 3 4 5 6 5 6 3 7 7 4 5 5 5 5 4 1 4 1 4 2 3 3 2 3

n13 4 2 3 4 3 5 2 6 4 5 4 6 3 7 7 4 6 5 5 5 1 1 3 4 3 4 2 3 4 5

n14 6 7 1 6 3 1 4 2 4 5 6 3 4 5 7 4 5 3 4 4 5 3 2 4 2 3 5 5 3 4

n15 5 4 4 6 3 4 4 6 5 5 4 5 4 5 7 3 2 4 6 3 4 1 3 5 2 7 1 3 3 2

n16 6 3 4 3 3 5 4 4 5 7 6 4 2 6 7 4 5 3 5 2 5 4 1 3 4 1 3 2 5 4

n17 5 3 4 2 1 5 3 4 6 5 4 7 4 6 7 5 4 6 5 5 3 2 4 1 3 3 2 4 4 3

n18 5 2 3 5 1 4 3 2 4 5 3 6 5 6 7 4 4 5 6 7 5 2 4 4 3 3 1 4 4 3

n19 4 6 6 3 2 7 5 4 7 5 3 3 4 3 2 4 4 4 4 3 5 2 5 5 5 6 1 4 1 3

n20 4 2 1 3 3 4 3 2 5 5 5 5 7 3 7 6 4 3 4 6 5 4 5 6 2 4 3 4 4 1

n21 2 5 5 4 3 3 4 5 5 7 2 1 4 3 4 3 4 4 5 6 7 3 5 3 6 2 1 4 4 6

n22 4 5 4 2 3 4 2 4 3 6 5 6 3 5 7 7 6 4 5 4 3 3 4 5 3 1 1 2 4 5

n23 4 5 5 1 2 4 2 1 4 7 3 3 4 4 2 4 5 3 6 6 5 4 7 4 6 5 5 3 3 3

n24 3 5 5 4 2 6 2 3 6 7 5 5 5 4 7 3 1 3 3 2 5 1 4 4 3 6 4 4 4 4

n25 3 6 2 3 5 3 4 4 7 6 4 2 5 5 6 5 5 4 3 2 1 4 4 7 3 4 3 1 4 5

n26 4 3 3 5 4 2 6 4 5 6 6 7 3 5 7 4 4 3 5 5 4 2 3 3 4 1 1 4 2 5

n27 4 5 3 2 4 3 3 4 5 7 7 5 5 5 6 5 6 3 3 4 4 1 2 6 4 2 1 3 4 4

n28 4 4 4 4 1 5 1 3 6 7 7 3 4 4 6 5 2 5 5 5 6 2 2 3 3 4 3 3 4 5

n29 4 2 1 5 3 6 3 2 4 6 6 4 5 5 7 5 5 5 7 4 3 2 4 3 3 4 1 3 4 4

n30 4 7 1 3 3 5 1 4 5 6 5 5 6 6 7 3 4 4 4 5 4 3 2 3 2 2 4 5 4 3

n31 5 5 3 4 2 3 4 1 6 7 7 5 5 4 6 4 5 5 3 6 4 2 4 4 2 1 3 3 4 3

n32 5 7 3 1 4 3 5 1 6 5 5 6 5 4 7 4 6 3 4 4 4 3 3 4 2 2 2 3 4 5

n33 4 7 6 3 4 3 3 4 6 7 6 3 4 5 5 5 4 2 2 2 4 3 1 1 5 4 5 3 4 5

n34 3 3 2 2 1 6 3 3 5 7 5 4 4 4 7 5 4 5 5 6 6 2 4 4 4 3 1 3 4 5

n35 2 6 2 7 4 3 4 3 5 6 7 5 4 4 6 5 5 3 3 5 3 1 1 4 2 3 4 4 4 5

n36 5 1 1 3 2 5 3 4 5 6 5 7 5 6 7 4 5 6 4 4 2 3 4 3 3 3 2 4 4 4

n37 2 5 2 1 5 4 3 1 3 4 6 3 7 4 6 5 7 5 4 4 4 4 5 5 3 3 4 3 2 6

n38 5 7 1 3 4 1 3 2 5 6 4 6 4 6 7 4 5 5 4 4 2 3 3 5 4 3 2 5 3 4

n39 5 5 2 3 2 3 1 5 3 6 7 6 5 4 7 6 4 5 5 4 3 4 4 3 1 2 4 3 4 4

n40 4 5 5 5 1 5 4 2 7 6 4 4 4 6 7 5 4 4 3 3 3 2 2 1 3 3 5 4 3 6

n41 4 6 7 5 1 4 3 4 5 6 5 6 5 5 7 3 4 4 4 3 3 2 1 3 2 2 3 4 4 5

n42 5 3 5 4 4 7 4 1 6 6 4 4 3 5 7 3 2 5 4 5 4 3 5 1 3 6 2 3 2 4
